# Geophysical early warning of salt precipitation during geological carbon sequestration

**DOI:** 10.1038/s41598-020-73091-3

**Published:** 2020-10-05

**Authors:** Ismael Himar Falcon-Suarez, Kurt Livo, Ben Callow, Hector Marin-Moreno, Manika Prasad, Angus Ian Best

**Affiliations:** 1 National Oceanography Centre, University of Southampton, Waterfront Campus, European Way, Southampton, SO14 3ZH UK; 2 Colorado School of Mines, Golden, CO 80401 USA; 3 University of Southampton, National Oceanography Centre Southampton, Southampton, SO14 3ZH UK; 4 Norwegian Geotechnical Institute, Ullevål Stadion, PB 3930, 08906 Oslo, Norway

**Keywords:** Climate sciences, Environmental sciences, Solid Earth sciences, Engineering

## Abstract

Sequestration of industrial carbon dioxide (CO_2_) in deep geological saline aquifers is needed to mitigate global greenhouse gas emissions; monitoring the mechanical integrity of reservoir formations is essential for effective and safe operations. Clogging of fluid transport pathways in rocks from CO_2_-induced salt precipitation reduces injectivity and potentially compromises the reservoir storage integrity through pore fluid pressure build-up. Here, we show that early warning of salt precipitation can be achieved through geophysical remote sensing. From elastic P- and S-wave velocity and electrical resistivity monitoring during controlled laboratory CO_2_ injection experiments into brine-saturated quartz-sandstone of high porosity (29%) and permeability (1660 mD), and X-ray CT imaging of pore-scale salt precipitation, we were able to observe, for the first time, how CO_2_-induced salt precipitation leads to detectable geophysical signatures. We inferred salt-induced rock changes from (i) strain changes, (ii) a permanent ~ 1.5% decrease in wave velocities, linking the geophysical signatures to salt volume fraction through geophysical models, and (iii) increases of porosity (by ~ 6%) and permeability (~ 7%). Despite over 10% salt saturation, no clogging effects were observed, which suggests salt precipitation could extend to large sub-surface regions without loss of CO_2_ injectivity into high porosity and permeability saline sandstone aquifers.

## Introduction

Deep siliciclastic saline aquifers are among the preferred options for geological CO_2_ sequestration (GCS), because of their low reactivity to CO_2_ and high storage capacities^1^. The storage efficiency is determined by the porosity (reservoir capacity), while the effectivity of the injection is controlled by the permeability (connectivity of the effective pore volume). Any changes in the storage capacity and/or injection efficiency during GCS activities may compromise the integrity of the reservoir^2,3^, and hence needs further study for long-term and safe sequestration of CO_2_.

The CO_2_ injected in a reservoir advances as a plume by displacing a fraction of the resident brine^4,5^. The injected CO_2_ partially dissolves in the parent brine while inducing evaporation of water. These two mechanisms, together with capillary pressure gradients towards the injection source, molecular diffusion of saline ions within the brine, CO_2_ gravity (buoyancy) and self-enhancing salt crystallization phenomena, can trigger complex salt precipitation patterns in porous media^6^. CO_2_-induced salt precipitation can lead to a significant decrease of up to 15% in porosity, and up to 85% in permeability^7^. Eventually, this phenomenon can dramatically impact the injection efficiency, as observed off-shore in the Tubåen Formation at the Snøhvit Field^8^.

Several studies have addressed the mechanisms and location of salt precipitates in saline aquifers during CO_2_ storage, and the negative consequences for the injectivity^9–14^. Numerical simulations predict significant salt-induced pressure build-up around the CO_2_ injection well, the preferential salt precipitation area^15–17^, even for high permeability rocks and low injection rates^18,19^. Experimental studies have confirmed CO_2_—induced salt precipitation in porous media from lab-on-chip experiments^6,20^ and core scale flow-through tests^21–23^. Experimental observations show that the precipitation process starts after a transition period post—CO_2_ injection, forming early pore scale crystals in the brine, followed by late polycrystalline aggregates at the CO_2_—brine interface^7^. The velocity and extent of the process depend on CO_2_ injection flow rate^18^, brine mobility^24^, pore network geometry^19^, and the physical properties of both fluids defined by the temperature, pressure and composition^25,26^, particularly brine salinity^7^.

Simple engineering mitigation strategies can be applied in the reservoir to prevent the salt precipitation during CO_2_ injection activities in the field. For instance, fresh water-washing—a technique originally used in gas-producing wells to avoid salt clogging^16^ that has been proposed for GCS prior to CO_2_ injection^7,17,27^. Early detection of salt precipitation is crucial for timely mitigation strategies and ultimately for preserving reservoir integrity. However, this requirement contrasts with the lack of experimental and modelling studies aimed at the identification of CO_2_—induced salt precipitation from field-scale monitoring datasets, especially geophysical remote sensing.

We conduct CO_2_ flow-through tests, using a high porosity–permeability non-reactive sandstone to isolate the CO_2_-induced salt precipitation phenomenon. Here we integrate elastic and electrical resistivity measurements, X-ray micro-CT imaging, and rock physics modelling, to assess the potential of combined seismic and electromagnetic surveys for early detection of CO_2_—induced salt precipitation.

### Core flood experiments

**Figure 1:**
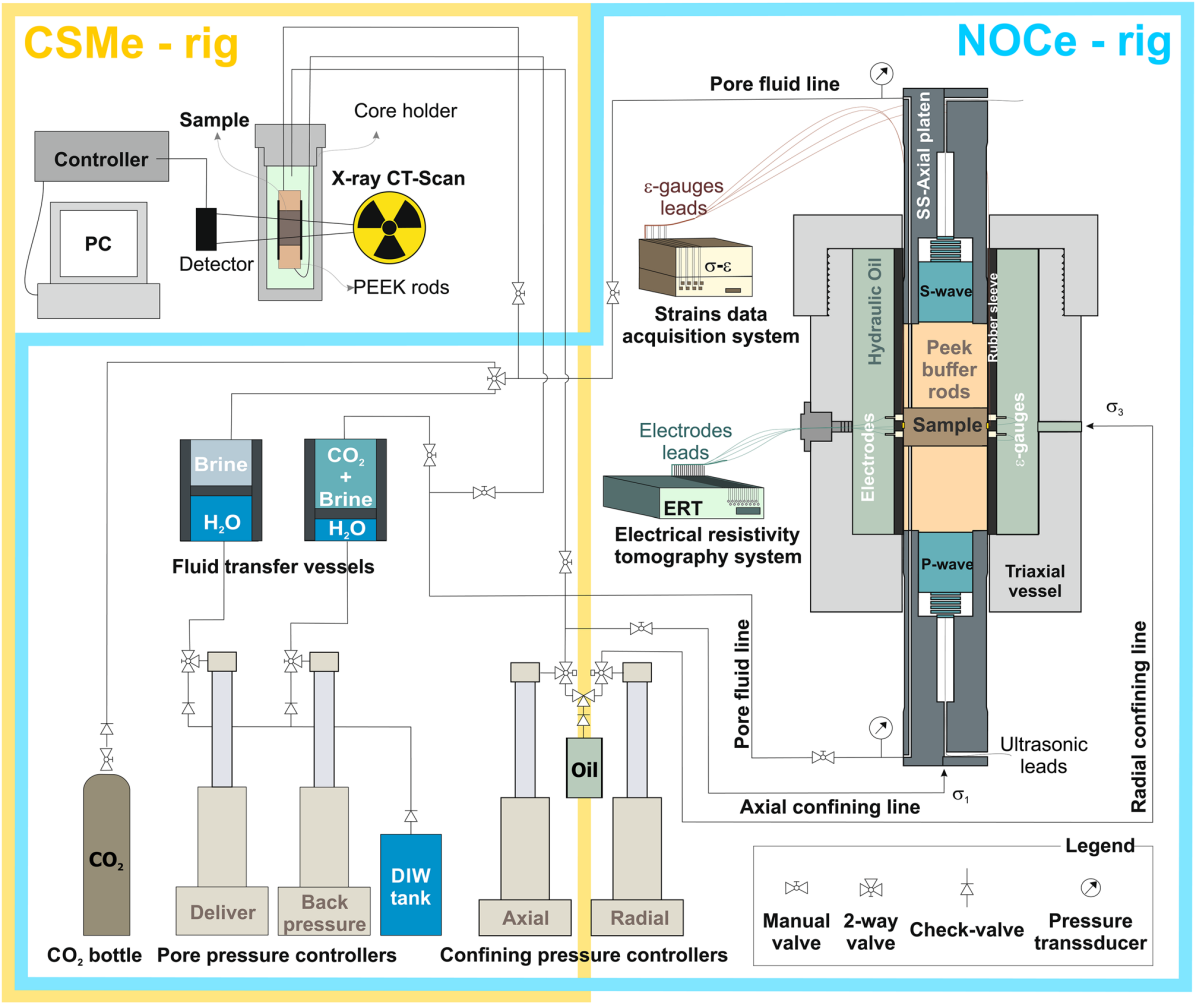
CSMe and NOCe experimental rigs.

This study involves two CO_2_ injection experiments (denoted as CSMe and NOCe) on a high porosity (~ 29%), high permeability (~ 1660 mD) synthetic quartz-rich sandstone, saturated with high salinity (X_NaCl_ = 25% wt. NaCl) synthetic brine. The experiments were conducted using two experimental setups for high pressure multi-phase flow-through tests (CSMe and NOCe rigs, Fig. 1), under constant hydrostatic confining pressure (P_c_ =  σ_1_ =  σ _2_ =  σ_3_ = 25 MPa), pore pressure (P_p_ = 5.5 MPa), and room temperature (~ 20 °C).

CSMe aimed at analysing the distribution of CO_2_, brine, and CO_2_-induced precipitation of salt crystals in the rock using micro-X-ray computed tomography (XCT) scans. In the first step, 17 pore volumes (PV) of brine were injected through the rock at a flow rate of ~ 0.2 cm^3^ min^-1^, followed by a XCT (lasting ~ 24 h). Next, CO_2_ gas (CO_2(g)_) was injected into the sample at ~ 0.2 cm^3^ min^-1^ continuously for 24 h (~ 200 PV), followed by a second XCT scan (~ 24 h). From the XCT, we developed a four-phase segmentation analysis to obtain the CO_2_, brine, quartz grain and salt volumetric fractions. Then, the sample was dried, cut and prepared into thin sections to compare with the original sample to assess the total salt content in the porous medium. Finally, the sample was subjected to X-ray diffraction (XRD) and scanning electron microscopy with energy dispersive spectroscopy (SEM–EDS) analysis to assess the precipitation of secondary minerals resulting from the CSMe test (see Supplementary Information). For the thin section analysis, we developed a three-phase (pore space, quartz grain and salt) segmentation analysis.

In the NOCe, we measured geophysical (ultrasonic compressional and shear, P and S, wave velocities and electrical resistivity) and hydro-mechanical (axial strains) properties, under three flooding conditions: (i) initial 25% NaCl brine flow-through the brine saturated sample (~ 10 PV); (ii) CO_2(g)_ injection flow displacing the brine from the saturated sample (drainage episode; ~ 75 PV); and (iii) 25% NaCl brine flow through the partially CO_2_ saturated sample (imbibition episode; ~ 30 PV). During the test, we alternated between two flow (Q) regimes: active measuring periods when Q was above 0, and interludes (Q = 0) without data collection. The test was repeated twice, named hereafter as NOCe Test-1 and Test-2, respectively.

## Results and discussion

### Salt saturation estimate (CSMe)

**Figure 2:**
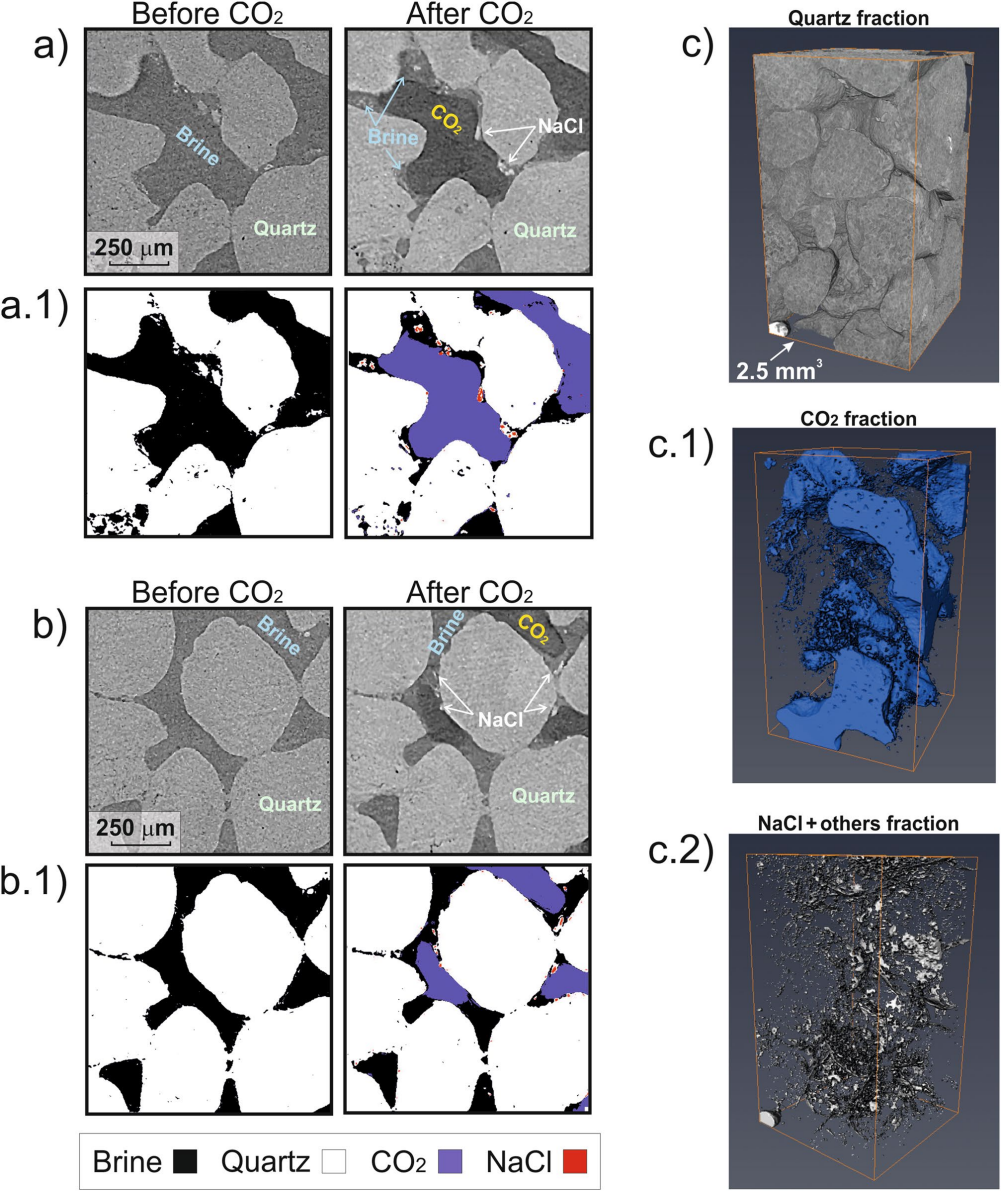
Salt precipitation evidence from the sandstone used in the CSMe. Examples of XCT scans before and after CO_2_ injection in 2D **(a, b)**, and 3D (**a** 2.5 mm^3^ volume portion) post-CO_2_ injection (**c** 1–3), including segmentation (**a.1, b.1**, **c.1–c.2**).

XCT image analysis (Fig. 2) shows that CO_2_ and brine occupied 42% and 50% of the total pore volume, respectively, after CO_2_ injection (i.e., second scan), yielding a salt saturation (S_NaCl_) of ~ 8% (Table 1). During the scanning the vessel was sealed, limiting the brine salinity to the dissolved ions within the pore water. Considering that the XCT scan lasted ~ 24 h, S_NaCl_ likely increased during scanning and therefore 8% represents a lower bound of S_NaCl_ in the CO_2_-brine-rock system after two days of CO_2_ exposure (i.e., one day of CO_2_ injection and one for the scan).

**Table 1:** XCT scan and thin section processed results.

CT-Run (CSMe)	Volume fractions (%)^a^				Saturation		
	CO_2_	Brine	Quartz	NaCl^b^	S_NaCl_	S_CO2_	S_brine_
Before CO_2_	0	29.7	69.6	0.7			
After CO_2_	12.7	15.1	69.0	3.2	0.08	0.42	0.50

Thin sections	Area fractions (%)						
	Pores	Grains	NaCl^b^	S_NaCl_			
Original sample	27.5 ± 0.5	71.4 ± 0.5	2.1 ± 0.2	0.0			
Fully dried after CSMe	22.5 ± 1.0	67.5 ± 1.0	10 ± 0.2	~ 0.19			

^a^General volume fraction error of 1% and saturation uncertainty of ± 0.01.

^b^NaCl phase contains NaCl plus others isotropic minerals.

**Figure 3:**
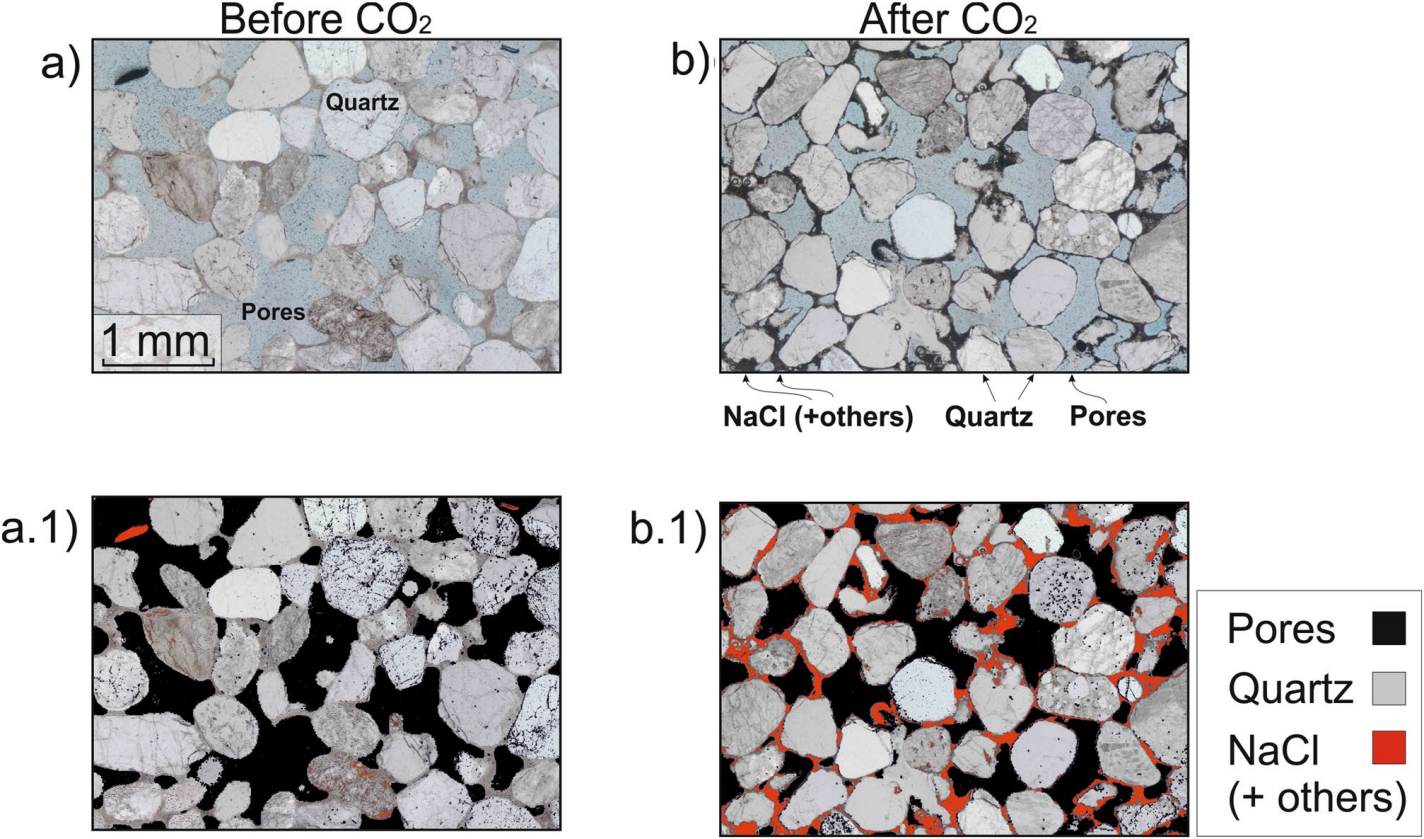
Salt precipitation evidence from thin sections of **(a)** the original sandstone used in the CSMe and **(b)** after full drying post-testing, including segmentation **(a.1, b.1)**.

**Figure 4:**
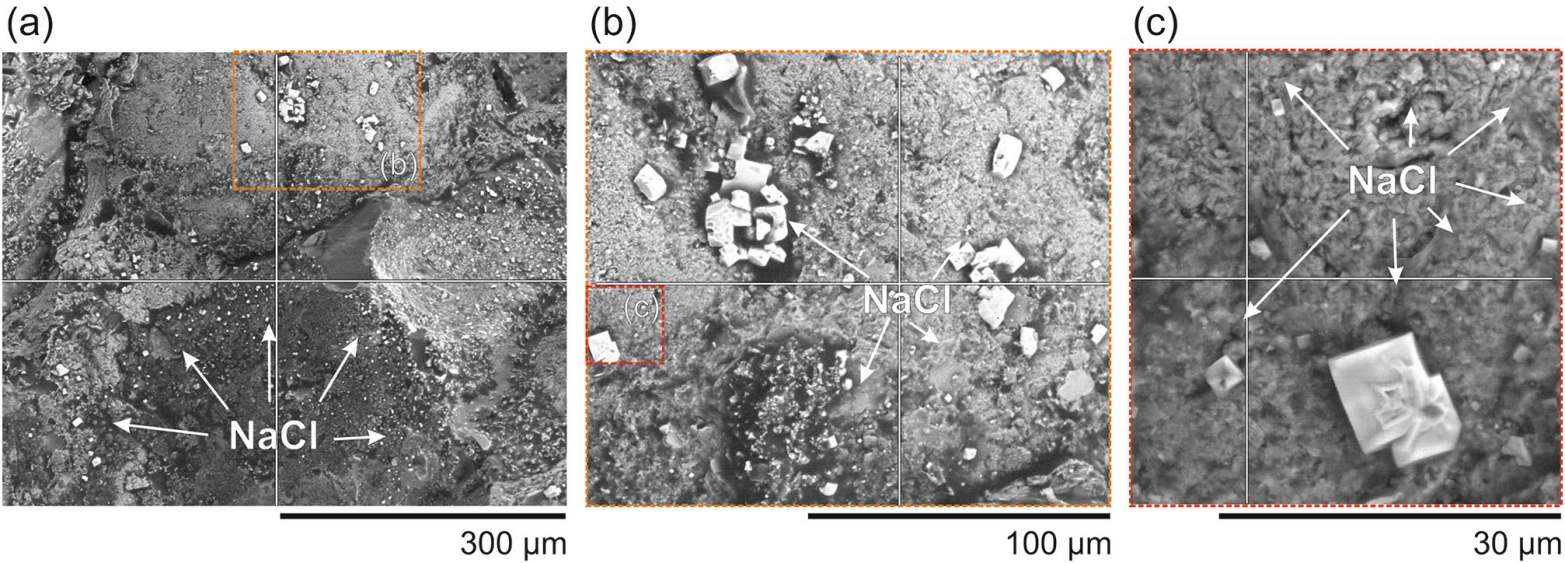
Scanning electron microscope (SEM) images showing salt crystals (NaCl) coating the original sample grains, in three zoom-in scales (from **a** to **c**), after full drying post-testing, corroborated by Energy Dispersive Spectroscopy (EDS) analysis (Supplementary Information).

Theoretically, using mass balance considerations, in our closed system, one single halite crystal of density ρ_NaCl_ = 2100 kg m^-3^ formed from the brine (S_brine_, with density ρ_brine_ = 1190 kg m^-3^ and salinity X_NaCl_ = 25% wt) occupying the available pore space post-test (i.e., 1 – S_CO2_) would fill 8.2% of the total pore volume (i.e., S_NaCl(theoretic)_ = (1—S_CO2_) · X_NaCl_ · ρ_brine_/ρ_NaCl_). However, thin section analysis (Fig. 3) reveals the subsequent drying process led to a final S_NaCl(observed)_ ~ 19% (± 2% derived from 2D-thin section to 3D transformation^28^). This analysis indicates that the salt precipitates around the rock grains mainly as an agglomerate of halite microcrystals, as previously observed^6^. This finding is corroborated by SEM–EDS analysis (Fig. 4) and XRD post-testing, which also shows NaCl crystals sometimes coexisting with nahcolite (NaHCO_3_) as subsidiary (further information in Supplementary Information). For practical reasons, we adopt the physical properties of the major component observed (i.e., halite) for the whole salt fraction and estimate a salt-aggregate micro-porosity of ~ 56.7% ± 2%, resulting from the ratio between theoretical and thin-section estimated S_NaCl_ post-test (ϕ_NaCl,micro_ = 1 – S_NaCl(theoretic)_/S_NaCl(observed)_). This micro-porosity fraction is below the image resolution for both the XCT and thin section processing. Accounting for this micro-porosity, the S_NaCl_ determined from XCT scan drops to 3.5%, indicating the salt precipitation was still in an early stage after two days exposed to CO_2_.

### Geophysical data analysis (NOCe)

**Figure 5:**
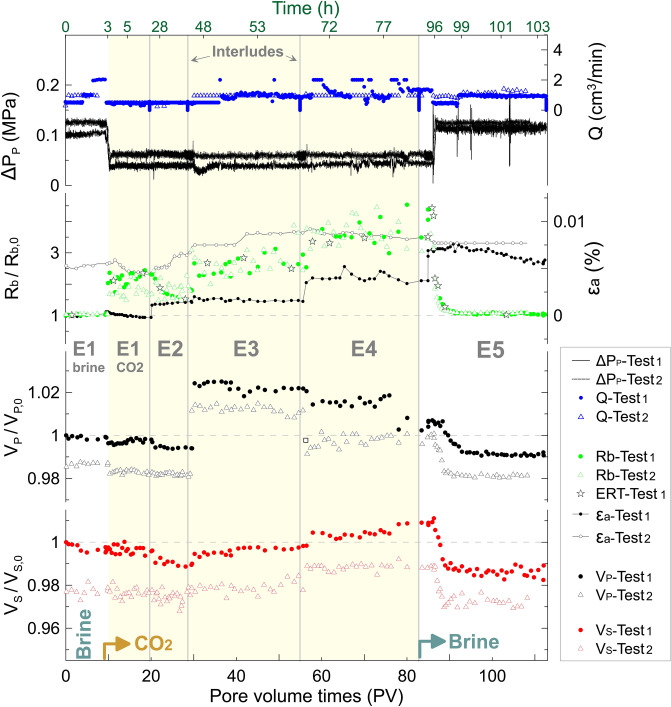
Results of two consecutive brine-CO_2_ flow-through tests (Test-1, black solid line and solid dots; Test-2, grey solid line and open triangles) in synthetic sandstone during the NOCe. Up- and downstream differential pore pressure (ΔP_p_) and total outlet flow (Q), P- and S-wave velocities (V_P_, V_S_), bulk electrical resistivity (R_b_), and axial strains (ε_a_ in %) are plotted versus pore volume (PV) and experimental time for three flooding episodes (E): brine flow (E1_brine_, below 9 PV), CO_2_ replacing brine (E1_CO2_ to E4; drainage), and brine replacing CO_2_ (E5; imbibition). Geophysical properties and strains are presented normalized with respect to initial values of Test-1. Solid vertical lines indicate interludes between episodes (i.e., Q = 0).

The NOCe CO_2(g)_ injection test lasted approximately five days (~ 115 h) and it was repeated twice, with an effective flow-through time of ~ 40 h that led to ~ 110 pore volumes (PV) throughput each (Fig. 5). Both tests show similar trends for all the measured properties except for the strains. Around 2% hysteresis effect of P- and S-wave velocities (V_P_ and V_S_) suggests a change in the physical properties of the sample during the experiment. On the other hand, the transport properties of the sample remained undiminished (under the experimental conditions), as indicated by an almost constant pore pressure gradient (ΔP_p_) throughout the NOCe for each pore fluid (i.e., brine flow in E1_brine_ and E5; CO_2_ from E1_CO2_ to E4) within the range of flow rates 0–2 cm^3^ min^-1^.

**Figure 6:**
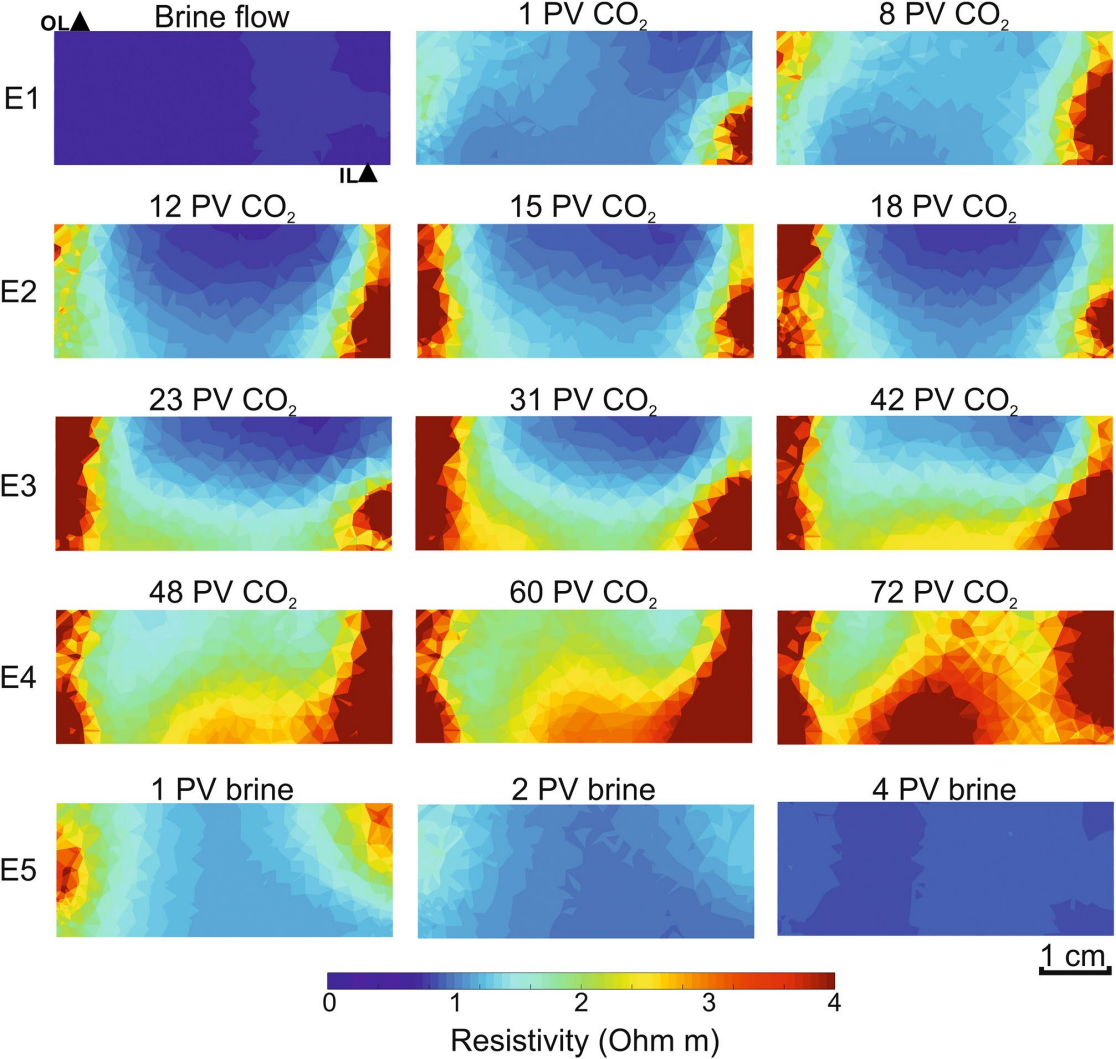
2D electrical resistivity tomography acquired during the NOCe Test-1 (star points in Fig. 5). The images are vertical planes crossing the centre of the sample, containing the inlet (IL) and outlet (OL) pore fluid ports as marked by solid triangles.

The CO_2_ arrival (episode E1_CO2_ after 9 PV of brine flow; Fig. 5), led to a resistivity increase of ~ 50%. Electrical resistivity tomography (ERT) images (Fig. 6) show preferential pathways localized peripherally by the inlet–outlet ports on the axial platens (diagonally opposite one other); the invariant differential pore pressure for changing flow-rates suggests the preferential pathways remained active through the test^29^. This annular fluid distribution provides very little variations on V_P_ and V_S_. Hence, the brine was first mainly drained from the edges of the sample whereas the CO_2_ is progressively saturating the resident brine towards the centre. Water evaporation rate into the CO_2_ stream is very low during this step^23^. This behaviour might indicate early salt precipitation acting as a lateral permeability barrier for CO_2_ advance towards the central part of the sample^18^.

After the initial gas-pushing water piston fluid substitution effect ^30^, each interlude leads to a redistribution of the two-phase fluids due to capillary effects that enhances salt precipitation^7,26^. During the interlude E1-E2, in the absence of effective pressure variations, the axial strain (ε_a_) increased (more significantly during the first test; Fig. 5). Since our quartz-rich sandstone is barely reactive to CO_2_, we interpret sample dilation as being caused by local salt-induced volumetric increase effects, previously observed in CO_2_-brine tests in sandstones under hydrostatic confining conditions^31^ and in air-brine systems under uniaxial loading^32^. In this regard, it has been recently demonstrated^33^ that salt crystals may exert pressures against pore walls up to ten times higher than the confining pressure used here (up to 150 MPa at the 50 μm observation microscale). Sample dilation continued during episode E2 accompanied by a weak decrease in both V_P_ and V_S_ by ~ 2%, and a resistivity increase by ~ 25%, in agreement with previously reported data^5,31,34–37^. Overall, E1 and E2 have similar pore fluid distributions (Fig. 6), and small geophysical variations, which can be explained by minor mechanical-chemical changes in the outer part of the sample.

The most significant changes in the elastic properties of the rock sample occur after the second interlude (from E2 to E3; Fig. 5), with V_P_ and V_S_ increasing by ~ 3% and V_S_ ~ 0.5%, respectively, in both tests. These changes contrast with the positive deformation of the sample (i.e., inflation) and the theoretical lowering of V_P_ for an increasing CO_2_ content^5,36^. However, our results agree with the scarce data reported about changes of elastic wave properties associated with salt precipitation in high porosity sandstone^8,38^. During E3 and E4, resistivity, strains, and V_S_ progressively increase while V_P_ decreases with CO_2_ injection. Despite a few sharp variations seen for all the parameters during the interlude E3–E4, E3 and E4 show similar trends that agree with those previously observed during CO_2_-brine drainage tests in sandstone^31,34,36,37,39^. During the last episode (E5, forced imbibition), the original brine flows through the sample, partially saturated in CO_2_. All the measured parameters recover to their original values except axial strains in barely five PV (Figs. 5, 6), a rapid recovery previously observed in CO_2_-brine-quartz systems under imbibition^31,35^. Resistivity totally recovered its original values. V_P_ and V_S_ carry ~ 1% of negative hysteresis at the end of the first test, and up to 1.5% after the second, with a minimum decrease from the end of Test-1 to start of Test-2. After the NOCe Test-2, the sample showed an increase of the original porosity, by ~ 6%, and permeability, by 7%.

### Saturation uncertainties

In a natural reservoir, a constant brine salinity scenario should be expected provided that salt clogging does not occur; in our test, the brine salinity progressively decreases as salt saturation increases. Since we calculate pore fluid saturations from the measured electrical resistivity and Archie’s laws^31,35^, we must first consider any changes in brine salinity caused by the CO_2_ drying process. The observed changes in bulk resistivity are mainly related to (i) a fluid substitution effect (i.e., high resistive CO_2_ displacing the original brine in the porous medium), and (ii) a progressive decrease of the original brine salinity with CO_2_ (i.e., ions-depleted residual brine due to salt precipitation). Each of these two factors reduces resistivity and affects the transformation of electrical resistivity into saturation differently.

Pore fluids saturation in a CO_2_-brine system can be calculated from electrical resistivity^31,34,36,39^, combining the first, ${\Phi}^{- \text{m}} = \frac{{\text{R}}_{\text{b,0}}}{{\text{R}}_{\text{w}}},$ and second, ${\text{S}}_{\text{w}}^{\text{n}} = \frac{{\text{R}}_{\text{w}}}{{\text{R}}_{\text{b,i}}}{\Phi}^{- \text{m} },$ Archie’s laws for fully and partially saturated porous media^40^, respectively. In these expressions, R_b_ is the bulk resistivity of the sample with subscripts 0 and i indicating full and partial saturation, R_w_ is the resistivity of the pore fluid, ϕ the porosity, S_w_ the brine saturation, and m and n are the cementation and saturation exponents, empirically derived for each particular rock.

The dissolved CO_2_ (less than 5% vol. at the experimental conditions^41,42^), is invisible to our resistivity and ultrasonic detection tools^35^, and therefore neglected in the saturation calculations based on Archie’s expressions above. This simple transformation is valid for invariable porous framework systems, i.e., in the absence of dissolution/precipitation phenomena. However, the XCT scanning showed a minimum S_NaCl_ (aggregate microcrystalline) of 8% for our experiments. CO_2_-induced salt growth progressively changes the bulk electrical properties of the rock, by changing both the porosity and brine resistivity. Therefore, to calculate S_w_, the combination of the first and second Archie’s laws has to be modified to account for each state of the rock with respect to the original values (subscripts i and 0, respectively), as follows:

1 $${\text{S}}_{\text{w}}^{\text{n}}\text{=}\frac{{\text{R}}_{\text{b,0}}}{{\text{R}}_{\text{b,i}}}\frac{{\text{R}}_{\text{w,i}}}{{\text{R}}_{\text{w,0}}}$$

In Eq. (1), we consider constant cementation (m) and saturation (n) exponents, because the salt aggregates observed in our CSMe test are suspended at the CO_2_-brine interface (Fig. 2). However, the cementation exponent is likely to increase in more advanced drying stages. Note that the change in water resistivity between stages is explicitly considered in Eq. (1), but the change in porosity due to salt precipitation is not. The micro-metre salt crystals are surrounded by highly conductive water films feeding the crystallization process^6^.

**Figure 7:**
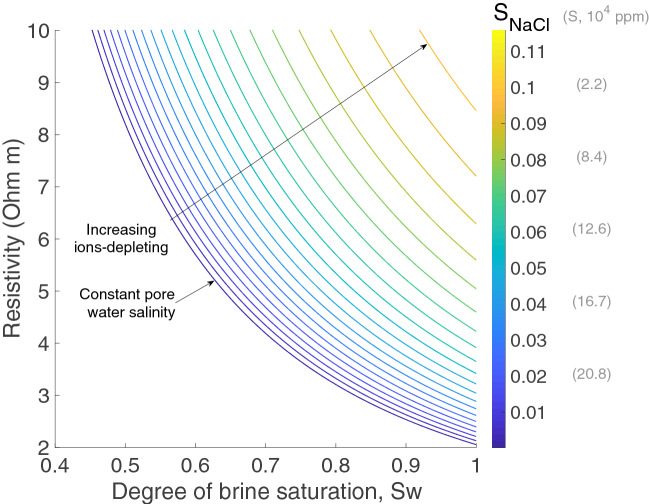
Bulk resistivity versus degree of brine saturation considering an initial brine salinity (S) of 25 × 10^4^ ppm, a constant temperature of ~ 20 °C, and the ions-depleting effect associated with the precipitation of salt (S_NaCl_, salt saturation) in a closed system.

At laboratory conditions (20 ºC), if we consider a closed system where all the original brine contributed its ions to halite formation, the *R*_*w*_ would have increased according to the expression^43^ ${\text{R}}_{\text{w}} = \left(4 \times 10^{5} / {\text{TS}}\right)^{0.88}$, with S being the brine salinity (in ppm) and T the temperature (in Fahrenheit). However, some backflow of brine towards the injection point^17^, particularly during interludes, might have partially contributed to salinity recovery. We consider the transition between a backflow completely renewing the resident brine (i.e., constant salinity), to no backflow leading to a progressively NaCl-depleted brine (Fig. 7). In our test, the degree of brine saturation estimated from bulk resistivity data should lie between both cases.

### Combining elastic and electrical properties

Salt can form either away from the mineral grains (non-cementing salt), or at grain contacts bridging mineral grains (cementing salt). Image analysis suggests a transition from non-cementing salt at early stages of salt formation (XCT images; Fig. 2) to cementing salt after fully drying (thin sections; Fig. 3). Early detection of this transition in the field using geophysical monitoring techniques is essential to initiate mitigation strategies in a timely manner, before both injectivity and ultimately the mechanical integrity of the reservoir are compromised (as suggested by the mechanical hysteresis observed in our data).

The bulk elastic properties of rocks are sensitive to the rock frame and pore fluid elastic properties^44^, and to the distribution of fluids in the porous medium for multiple fluids (e.g., uniform/patchy^45^). Hence, to infer such a transition in the salt distribution from our geophysical data, we can use the so called uncemented and cemented sand models^46,47^, and introduce them into Biot-Stoll poroelastic formulation^48–50^ to calculate frequency-dependent P and S wave velocity and attenuation. This approach has been applied previously to understand and assess quantitatively the impact of marine methane hydrate on these properties^51,52^. Due to the similarities in the distribution of hydrate and salt in the pores, we adopt these models using the physical properties (bulk and shear moduli, and density) of halite.

**Figure 8:**
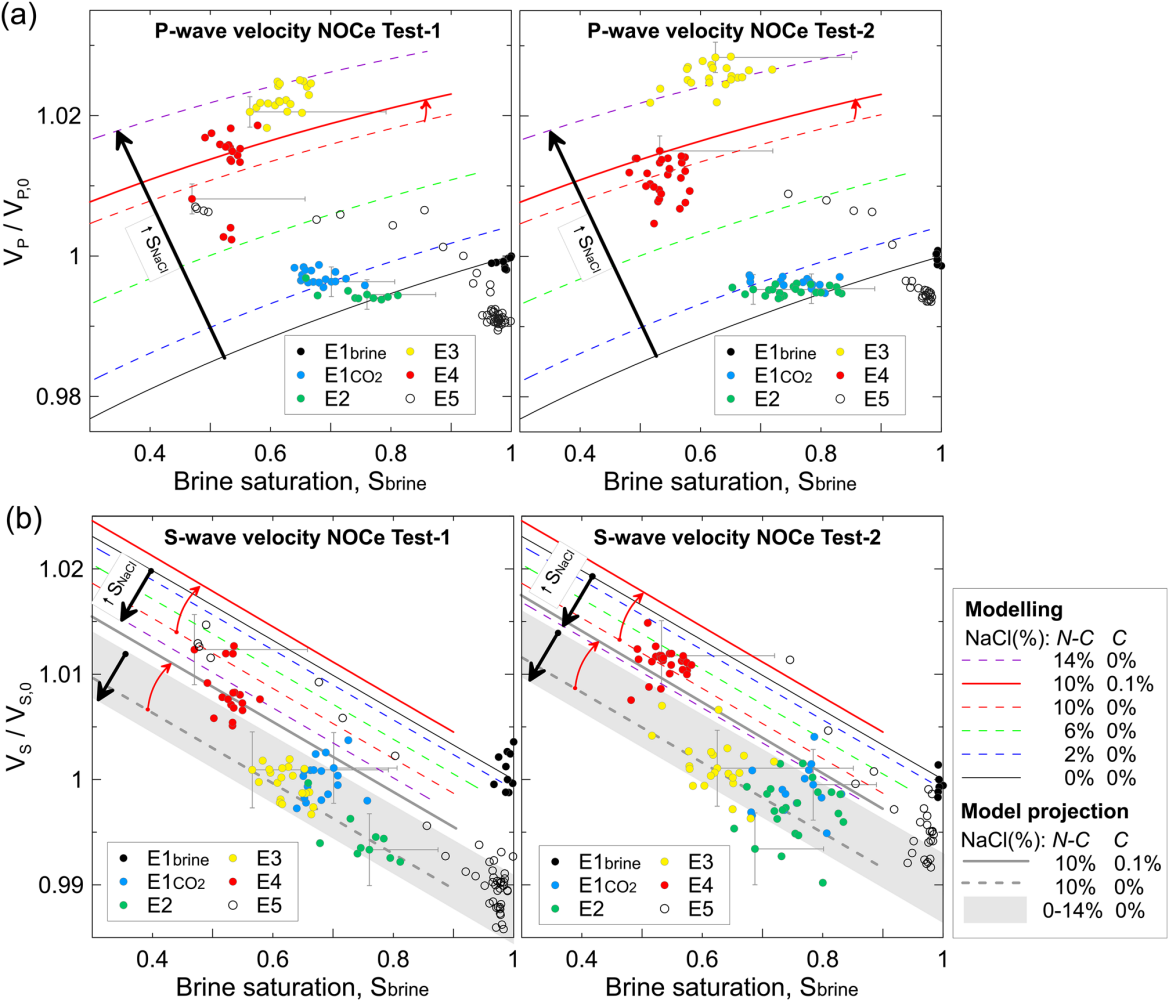
**(a)** Compressional and **(b)** shear ultrasonic wave velocity (V_P_ and V_S_), normalized with respect to their original brine saturation (subscript 0) values for NOCe Test-1 and Test-2, versus brine saturation, S_brine_, for the five episodes (E1–5) of both NOCe tests. The red arrow indicates the transition at S_NaCl_ 10% from non-cementing (N–C) to N–C with 0.1% of cementing salt (red model curves). To facilitate the analysis, we assume constant brine salinity of 25 × 10^4^ ppm (i.e., minimum S_brine_ possible; see Fig. 7), and add one cross error bar per episode to account (horizontally) for the effect of ions depletion during salt precipitation and (vertically) the uncertainty around the ultrasonic velocity determinations.

Using these model predictions, we can explain the observed evolution of the CO_2_-brine-salt-sandstone system from changes in the compressional and shear wave velocities with brine saturation, with both the NOCe Test-1 and Test-2 showing very similar results (Fig. 8). We assume that non-cementing salt only modifies the bulk modulus of the fluid. In contrast, cementing salt modifies both the shear and bulk moduli of the dry granular frame. Hence, V_S_ can be significantly affected by cementing salt but not by non-cementing salt (only minor changes can occur due to changes in bulk density of the rock).

For P-waves, the change in V_P_ from E1_brine_ (brine flow) to E1_CO2_ (CO_2_ flow) can be explained by the presence of up to ~ 4% of solid non-cementing micro-crystalline NaCl into the pore space during Test-1 (~ 2% for Test-2), partially reabsorbed during the interlude E1-E2 (Fig. 8a). The most significant change in V_P_ occurs from E2 to E3. During this interlude, the drying through evaporation is likely to be enhanced by capillary and diffusive mechanisms, leading to a higher crystallization rate^7,26^. The model indicates a sharp S_NaCl_ increase from E2 (S_NaCl_ ~ 1%) to E3 (S_NaCl_ ~ 13% in Test-1 and ~ 14% in Test-2). The brine remaining in the pore space is then NaCl-depleted and salt crystallization stabilizes, leading to a (CO_2_—brine) fluid substitution stage with some salt reabsorption phenomena at the end of the episode E4.

For S-waves, V_S_ reflects an early rock weakening effect during the transition from brine to CO_2_ injection (V_S_ drops from E1_brine_ to E1_CO2_), likely associated with poorly attached silica cement or grains, removed during early stages of the CO_2_ flooding due to sudden changes in the bulk properties (i.e., early salt nucleation) of the pore fluid (Fig. 8b). This early weakening effect is observed in both NOCe tests, but more significantly in Test-1. To isolate the CO_2_-induced salt precipitation effect after this early weakening, we project the modelling results to fit the data after E1_CO2_ (grey bands and grey lines in Fig. 8b) by considering the final V_S_ value of each test as V_S,0_. From E1_CO2_ to E3, the V_S_ data scatter (within the ultrasonic measurements error band) over the whole range of results for the non-cementing salt modelling (i.e., S_NaCl_ from 0 to 14%). The clear increase in V_S_ from E3 to E4 can be explained by considering a transition (red arrows in Fig. 8) from only non-cementing salt in E3, to non-cementing salt with a small fraction (0.1%) of cementing salt in E4 (red curve in Fig. 8); grey solid curve for the projection in Fig. 8b). Finally, brine is injected into the sample, and rapidly replaces (and dissolves) the salt-rich CO_2_-brine mixture in the pore space.

For a precise quantification of the evolution of the three pore phases, we would need to account for the progressive ions depletion of the original brine, which could be significant, as shown in Fig. 7; this would require monitoring of pore water conductivity during the CO_2_ flooding, which is missing in this experiment. Instead, we have added one single horizontal error bar to reflect the uncertainty associated with the increasing pore water resistivity for each episode (E1–E5).

The transition from non-cementing to cementing salt (E4) occurs after a substantial S_NaCl_ increase from E2 to E3 of (from ~ 2 to ~ 12% in Test-1 and up to ~ 14% in Test-2) well-defined by ~ 3% increase in V_P_ (Fig. 8a). This rapid S_NaCl_ increase occurs in the absence of CO_2_ flow (interlude E2–E3). By contrast, during flowing episodes, changes in rock properties are better explained by fluid substitution mechanisms as data points within each episode evolve towards higher CO_2_ saturations.

### Insights of salt distribution in geological reservoirs

From the constant response of our pore pressure sensors for different flow rate conditions all through the NOCe tests, we conclude that any clogging effects due to salt induced porosity reduction were insignificant. The distribution of gas-induced salt precipitation in a porous medium can be described through the Peclet (P_e_ = LV/D), and Damköhler (Da = Lk_r_ / V for Pe > 1^53^) numbers (reference length, L; effective flow velocity, V; diffusion coefficient of salt in water, D; kinetic rate constant, k_r_). Pe determines the importance of advective (Pe > 1) and diffusive (Pe < 1) transport^54^, while Da describes to which extent the dissolution/precipitation processes are dominated by fluid velocity or mineral reactivity^53^. For Pe >  > 1, salt precipitation would occur close to the injection zone, whereas Pe <  < 1 would lead to a homogeneous distribution^23^; the crystallization process is controlled by diffusion when Da >  > 1^55^. In our closed system, the calculation of Da is not straightforward, as it varies with the ions concentration decay in original brine with the increasing S_NaCl_. In the NOCe, the Pe changes from the order of 10^2^ (advective regime) during flowing episodes (i.e., V > 0), to null during interludes (i.e., V = 0), and the system is completely controlled by the diffusion (i.e., Da tends to infinity). Since this change in Pe is expected to enhance the homogenization of salt precipitation, it explains the S_CO2_ and S_NaCl_ backwards transition from E1_CO2_ to E2 during Test-1 (Fig. 8a).

Overall, upon detection of CO_2_-induced salt precipitation in a reservoir formation through seismic and electromagnetic geophysical surveys, reducing or stopping the CO_2_ injection is advisable for high porosity and permeability reservoirs and low brine salinity (e.g., Sleipner CCS field^35^). However, stopping or reducing the CO_2_ injection (i.e., minimum Pe; maximum Da), might have undesirable effects for reservoirs with lower porosity and permeability and higher brine salinity^8,56^. In these latter reservoirs, the higher capillarity of the rock could lead to self-enhancing salt growth phenomena^6^, rather than salt reabsorption and homogenization, which would extend the salt precipitation to large sub-surface regions. In turn, this would reduce the effectiveness of common clogging mitigation techniques, such as fresh water flooding, mainly focused on the surroundings of the injection well.

Furthermore, salt would be prone to fill (micro-) pores and start cementing the rock, thus modifying rock mechanical properties. During the GCS post-injection stage, natural aquifer recharge might lead to re-dissolution of salt crystals, this time with permanent changes in rock mechanical properties, as suggested by the hysteresis shown by the axial strain (only ~ 5% recovery) during the NOCe, together with the V_P_ and V_S_ drop and the increase of the original porosity and permeability, during NOCe Test-1 and Test-2.

## Conclusions

During a set of CO_2_ flow-through experiments using a synthetic sandstone of well-known petrophysical properties, we have found evidence of CO_2_-induced salt precipitation from X-ray micro-CT imaging and of detectable geophysical signatures (elastic waves and electrical resistivity) associated to this phenomenon. Our results show for the first time how elastic wave velocities, when combined with electrical resistivity measurements, can be used to detect early stage salt precipitation during CO_2_ injection into reservoir sandstones. Based on our experimental results, we conclude:

1. The precipitation of salt induced by CO_2_ injection into brine saturated sandstones significantly affects measurable geophysical properties of the original rock formation, i.e., compressional and shear wave velocity, and electrical resistivity. During early stage injection, salt precipitates away from grain contacts (non-cementing), then starts to cement the rock grains after a certain threshold salt saturation is achieved (10% in our experiment).
2. The precipitation of salt triggers detectable changes in compressional wave velocity (up to 3%) that can be differentiated from pore fluid substitution effects with the aid of electrical resistivity, based on the latter’s sensitivity to electrically insulating gas phases like CO_2_. While compressional wave velocity is sensitive to both non-cementing and cementing salt precipitation, shear waves velocity is predominantly sensitive to the fraction of cementing salt.
3. During CO_2_ -induced salt precipitation, pore fluid salinity changes have to be considered to successfully estimate the evolution of CO_2_, brine and salt saturations from electrical resistivity.
4. CO_2_-induced salt precipitation may lead to dilation of the rock frame, even in high porosity and permeability sandstones that are barely reactive to CO_2_. Hence, early detection of salt precipitation is crucial to preserve reservoir injectivity properties and mechanical integrity.

## Materials and methods

### Rock samples and pore fluid

We created a homogeneous synthetic sandstone of high (effective) porosity (ϕ = 0.29; He-pycnometry), and high absolute permeability k_abs_ ~ 1660 mD (N_2_-permeameter), to avoid salt-induced clogging during CO_2_ injection. Following the manufacturing procedure described in Falcon-Suarez, et al.^57^, we used sorted, coarse grained (diameter > 500 µm) sand and low cement-to-grain ratio to ensure high porosity and permeability (with a final dry density ρ_d_ ~ 1826 kg m^-3^) and to avoid clay conductivity and clogging effects. The composition of the sample was ~ 97% quartz, ~ 3% albite (X-ray diffraction, XRD, analysis with a Philips X’Pert pro XRD-Cu X-ray tube), which ensures the applicability of the original Archie’s laws without applying corrections related to the presence of clay minerals in the rock. From the original sample, we extracted two core plugs of (sample A) 2 cm length, 1.2 cm diameter, and (sample B) 2 cm length, 5 cm diameter, for the CSMe test and for the NOCe tests, respectively.

We used a 25% NaCl synthetic brine solution prepared with deionized water in both tests, keeping NaCl concentration slightly below the saturation point to promote rapid crystallization during CO_2_-induced evaporation. To ensure the saturation condition, the samples were first oven-dried, then saturated with the experimental brine via imbibition under vacuum conditions, and finally flushed at 5 MPa with the experimental brine solution to enhance the dissolution of remaining air in the system. At the pore pressure used in this test, the salinity of the brine has neglectable effect on the mutual solubility of CO_2_ and brine.

### CSMe setup and test configuration

The brine saturated sample A contained in an in-house developed Aluminium pressure vessel was pressure equilibrated for one night at the target P_c_ (25 MPa) and P_p_ (5 MPa) conditions, and left for one night for stress–strain equilibrium. Then, the test started with a brine flow episode at 0.2 cm^3^ min^-1^, up to completing 17 PV of brine flow-through. Immediately after, the first XCT-scanning was carried out lasting ~ 24 h. Next, CO_2_ was injected in the sample at variable flow rate (0.1—0.2 cm^3^ min^-1^) continuously during 24 h (Peclet’s number Pe ~ 60), reaching ~ 200 PV of collected pore fluid volume downstream. Immediately after, the second XCT-scanning was conducted.

The pressure vessel setup consisted of an aluminium tube sealed with commercial high-pressure fittings, and three flow-through lines for confining, and inlet and outlet pore pressure. The sample A was jacketed and plugged with in-house manufactured endcaps, allowing pore fluid flow-through while isolating the sample from the confining hydraulic oil. Pressure vessel connecting lines were flexible to ensure free twisting of the vessel within the XCT instrument during XCT image acquisition. We performed the measurements using a Xradia Micro XCT-400. Tomographic images were collected in increments of 0.1 degrees of rotation with a background reference image generated from 10 averaged individual scans preceding installation of the pressure vessel. The reference image was subtracted from the measurement images to give adjusted values for the sample and vessel without background effects. Optimal tomography angles were calculated using specialist software XM-Controller 8.1.7546 (XRadia Inc.; https://www.zeiss.com/microscopy/int/products/x-ray-microscopy), and measurements were obtained with 0.5 × and 4 × lens yielding resolutions of approximately 25 and 3 microns, respectively. For this study, we only used the latter (high resolution image set). Tomographic images were generated following beam hardening and centre shift corrections to ensure optimal image quality in the reconstructed images, using Avizo software version 9.5.0 (https://www.fei.com/software/avizo-user-guide). The XCT-images were segmented using a 3D weka semi-automated segmentation^58^ to characterize the four phases of interest: CO_2_, brine, quartz and the rest, which included ore minerals and salt (Fig. 2). First, we selected a sub-sample (~ 1.5 mm^3^) and obtained the four phases; second, we extrapolated the results to the whole sample, following the procedure described in Callow, et al.^59^.

After the test, the sample was left to dry under atmospheric conditions. Then, thin sections were obtained from both the original and the tested samples, to assess the total salt content in the porous medium. The images were analysed using Fiji-ImageJ software version 2.0.0-rc-68/1.52 g (https://fiji.sc) to measure the total salt in the sample. The blue-resin method used to make the thin sections allowed the segmentation of the obtained in plane-polarized light images into four domains: pores (blue), grains (light grey), cement (brownish), and salt (black regions: isotropic salt crystals and ore minerals). The processing consisted of a threshold filtering to define the pores (ϕ) and salt (S) fractional areas, and then transform it to grey scale to calculate the salt saturation as S_NaCl_ = S / (ϕ + S).

### NOCe setup and test configuration

The rig is configured around a triaxial cell core holder under accurate control (ISCO pumps) of confining and pore pressure^31^. The rubber sleeve inside the vessel has 16 stainless steel electrodes connected to an electrical resistivity tomography data acquisition system^31,35^. Using a tetra-polar electrode configuration, each run collects 208 individual (tetra-polar) measurements, which are then inverted using a variation of the software EIDORS (version 3.1) MATLAB toolkit (https://eidors3d.sf.net). Axially, two (confining) platens house ultrasonic pulse-echo sensors, isolated from the sample and the rest of the rig by two polyether ether ketone (PEEK) buffer rods of well-defined acoustic impedance and low energy loss. This configuration provides a reliable delay path within the frequency range 400–1000 kHz, which enables the identification of top/base sample reflections for calculating ultrasonic P- and S-wave velocities and attenuations using the pulse-echo technique^18,19^, with ± 0.1% velocity precision and ± 0.3% accuracy (95% confidence), while up to ± 5% for the attenuation. The signals are displayed on a digital oscilloscope LeCroy Wavesurfer 4000HD (https://teledynelecroy.com/) and stored on the computer for spectral analysis assembly using MATLAB R2017a (https://www.mathworks.com). For this test, we processed the ultrasonic data from Fourier analysis of broad band signals to compare the ultrasonic properties of our three samples at a single frequency of 550 kHz.

The sample B was equipped with 350 Ohms electrical strain gauges, epoxy-glued on the lateral side of the sample, to record axial (ε_a_) strains during the tests in continuous (every two seconds), with an accuracy of ± 10^–6^ m m^-1^ (i.e., < 1.5% error for the deformation range in our experiment; Fig. 5). The strain gauges limited the ERT interpretation to the vertical plane (i.e., 2D) elongated 90° with respect to the gauges position. Along with the strains, the pore pressure was also monitored with two additional pressure (piezoresistivity) sensors located directly up- and downstream of the sample. The injection of both the brine and CO_2_ were carried out under controlled pore pressure downstream (5.5 MPa) and outlet flow. To avoid early compaction effects, the sample was initially placed into the triaxial vessel at the test conditions during four days, and then subjected to minimum brine flow-through (0.01 cm^3^ min^-1^). Injected confining fluid and the strains indicated mechanical stabilization occurred after two days. After the first test, the confining and pore pressure were dropped by keeping constant the differential pressure (P_diff_ = P_c_ – P_p_ = constant, to minimize stress-induced damages), down to atmospheric P_p_ conditions. Then, first, the sample was newly saturated via vacuum imbibition under confining inside the vessel for ~ 3 h; next, under continuous brine flow, both P_c_ and P_p_ were again increased at constant P_diff_, first up to P_p_ = 10 MPa (i.e., P_c_ = 30 MPa) during 1 h flow to enhance dissolution of any remaining CO_2_ bubble, and finally to the original test conditions (P_c_ = 25 MPa and P_p_ = 5 MPa). Once at the test conditions, the CO_2_ flow-through test was repeated, replicating the episodes of the first one. After the NOCe Test-2, the sample was batched in DIW for a week, and then dried to measure the final (subscript f) porosity (ϕ_f_ = 0.306; He-pycnometry) and permeability (k_abs,f_ ~ 1771 mD; N_2_-permeameter).

### Elastic wave modelling: rock with salt in the pores

Our modelling of elastic wave parameters follows Ecker, et al.^51^ approach that considers two idealized models for solid phase precipitation in the pores (gas hydrate in their case and salt in this work). We combine the stiff uncemented and cemented sand models^46,47^ using MATLAB (R2017a) to calculate the frame properties of the dry rock, and applied Biot-Stoll^48–50^ to calculate P and S-wave velocity and attenuation over the whole brine saturation range at a frequency of 550 kHz. Fluid density, viscosity and compressibility at the experimental pressure, temperature and salinity conditions were calculated using the High Pressure International EOS for brine^60^ and data from the National Institute of Standards and Technology (https://webbook.nist.gov/chemistry/fluid/) for CO_2_.

## Supplementary information

Supplementary Information.

## Data Availability

Data presented in this study will be publically available: (1) the XCT data (CSMe tests) at the OSF (https://osf.io/) and the Colorado School of Mines website (https://crusher.mines.edu/publications/); and (2) the geophysical data (NOCe tests) at the UK National Geoscience Data Centre (NGDC) repository (https://doi.org/10.5285/6c9d05aa-f1f2-49a9-868e-3d2d5947ad54).
