# Supplementary material for: Geophysical early warning of salt precipitation during geological carbon sequestration
Source: Sci Rep. 2020 Oct 5;10:16472. doi: 10.1038/s41598-020-73091-3 (PMC7536243; doi:10.1038/s41598-020-73091-3)
Supplement: Supplementary file 1 — Supplementary Information. [file 41598_2020_73091_MOESM1_ESM.pdf]

Supplementary Information for:

Geophysical early warning of salt precipitation during geological carbon sequestration

Ismael Himar Falcon-Suarez[1]*, Kurt Livo[2], Ben Callow[1,3], Hector Marin-Moreno[1,4], Manika Prasad[2] and Angus Best[1]

[1] National Oceanography Centre, University of Southampton Waterfront Campus. European Way, SO14 3ZH, Southampton, United Kingdom.
[2] Colorado School of Mines, Golden, Colorado 80401, USA
[3] University of Southampton, National Oceanography Centre Southampton, Southampton, SO14 3ZH, UK
[4] Norwegian Geotechnical Institute, PB 3930 Ullevål Stadion, NO-08906 Oslo, Norway.

XRD and SEM-EDS analysis

The sample used in the CMSe test was subjected to X-ray diffraction (XRD) and Scanning Electron Microscopy (SEM - Carl Zeiss Leo 1450VP) with Energy Dispersive Spectroscopy (EDS - Oxford Instruments X-Act detector with a 10 mm^2 detector window; software AZtec v.3.3) analysis after the CO_2 flow-through test to assess the resulting mineralogy.

The XRD study focused on identifying mineralogy of the precipitated crystals. The data show a reasonable fit for the 100% halite (NaCl) peak (Figure S1), while the presence of nahcolite ($NaHCO_3$) remains inconclusive as its peak overlaps with that of the halite with potassium.

The SEM-EDX results support the occurrence of sodium bicarbonate and/or another Na-bearing and Cl-poor mineral phase. For this analysis, the sample was mounted on an SEM stub after being Au-coated. Figure S2 shows a low magnification image of a quartz grain with regions of increased back scatter signal (white-light grey tone). EDS analyses were taken from the yellow/orange areas (i.e., s33 to s38). The data from these analyses are included in Table S1.

The regions with a cleaner Na and Cl signal consistently come up with differing weight percentages for each element. Removing and correcting for minor elements we find that the atomic weights of Na and Cl are present Na:Cl ratios of around 50:50 (i.e., NaCl), and 58:42, which indicates preferable formation of a more Na-rich phase or mix of phases.

A line scan across one region with an atomic ratio corresponding with NaCl (in spectrum area s35 in Figure S2; Table S1) and into another that did not, shows that the atomic ratio changes from approximately 50:50 to 58:42 (Figure S3). This would support the hypothesis that halite and sodium bicarbonate (nahcolite) can co-precipitate. However, the persistently high Cl value within the hypothesized halite/ nahcolite mix suggests that nahcolite is subsidiary to halite, which is also supported by the stronger XRD signal for halite and only a possible signal for nahcolite.

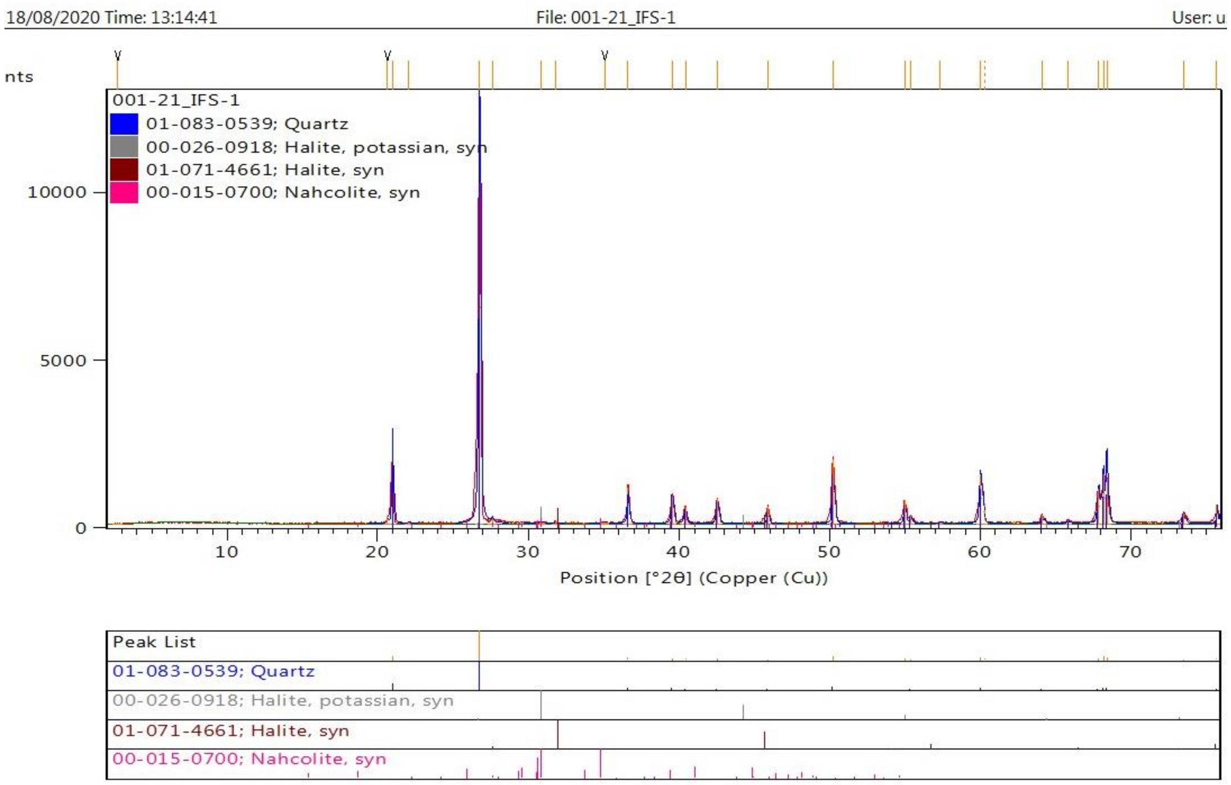

Figure S1. X-ray diffraction (XRD) analysis on the sample used for the CMSe test.

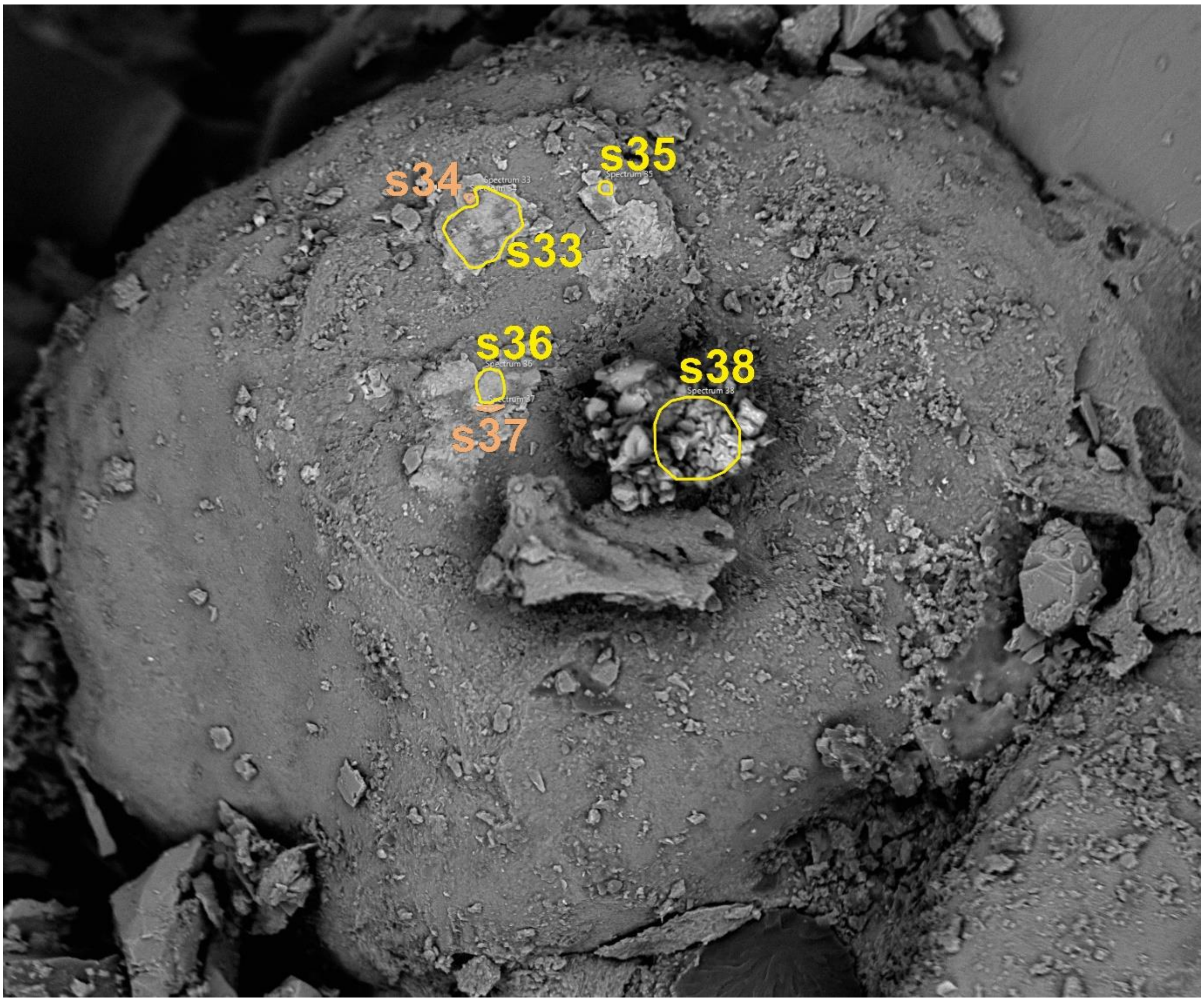

Figure S2. Quartz grain covered by salt aggregates. Regions s33 to s38 denote the areas from where EDS analyses were conducted.

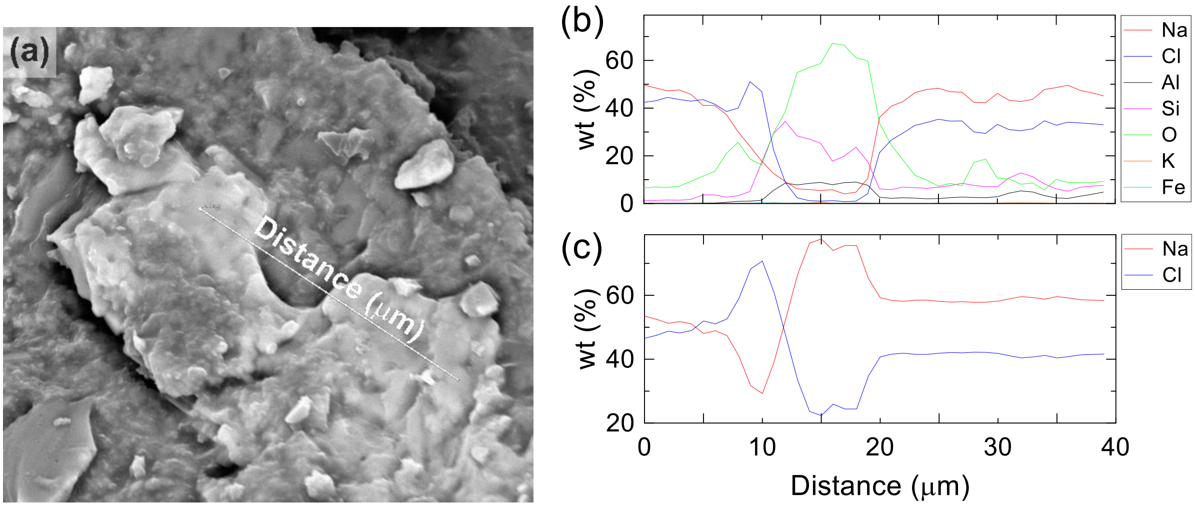

Figure S3. Atomic weight analysis from the spectrum s35 (Figure S2; Table S1). (a) Scan line; (b) general atomic weights; (c) normalized atomic weights for Na and Cl.

Table S1. EDS analyses from the regions marked in Figure S2

Spectrum	Elements	wt%	wt% Sigma	Atomic %
s33	C	44.61	1.59	59.99
	O	18.39	0.7	18.57
	Na	16.06	0.48	11.28
	Al	1.3	0.06	0.78
	Si	3.65	0.12	2.1
	Cl	15.99	0.47	7.28
s34	C	50.64	1.48	67.66
	O	9.44	0.57	9.47
	Na	19.92	0.61	13.91
	Al	0.31	0.03	0.19
	Si	1.31	0.05	0.75
	Cl	17.58	0.53	7.96
	Pb	0.79	0.17	0.06
s35	C	36.17	2.99	56.35
	O	5.65	0.59	6.61
	Na	23.68	1.12	19.27
	Al	0.18	0.04	0.13
	Si	1.19	0.07	0.8
	Cl	31.69	1.5	16.73
	Pb	1.43	0.25	0.13
s36	C	45.06	2.02	63.57
	O	8.99	0.62	9.52
	Na	18.69	0.7	13.78
	Al	0.98	0.06	0.62
	Si	3.25	0.13	1.96
	Cl	21.87	0.81	10.46
	Pb	1.16	0.21	0.09
s37	O	7.27	1.42	20.41
	Na	2.19	1.07	4.27
	Si	2.83	0.39	4.53
	Cl	0.6	0.28	0.76
	Fe	87.1	1.7	70.03
s38	C	55.5	3.82	64.73
	O	34.7	3.44	30.38
	Si	9.8	0.86	4.89
